# A stomata imaging and segmentation pipeline incorporating generative AI to reduce dependency on manual groundtruthing

**DOI:** 10.1186/s13007-025-01451-z

**Published:** 2025-11-13

**Authors:** Changye Yang, Huajin Sheng, Kevin T. Kolbinson, Hamid Shaterian, Paula Ashe, Peng Gao, Wentao Zhang, Teagen D. Quilichini, Daoquan Xiang

**Affiliations:** 1 Aquatic and Crop Resource Development Research Centre, National Research Council Canada, 110 Gymnasium Pl, Saskatoon, S7N 0W9 SK Canada; 2 Department of Biology, University of Saskatchewan, 112 Science Pl, Saskatoon, S7N 5E2 SK Canada; 3 Saskatoon Research and Development Centre, Agriculture and Agri-Food Canada, 107 Science Place, Saskatoon, S7N 0X2 SK Canada

**Keywords:** Stomatal traits detection, Machine learning, Generative AI, Synthetic data

## Abstract

Stomata regulate gas and water exchange in plants and are crucial for plant productivity and survival, making their trait analysis essential for advancing plant biology research. While current machine learning methods enable automated stomatal trait extraction, existing approaches face significant limitations that require extensive manual labeling for training and additional human annotation when applied to new species. This study presents an automated system for extracting stomatal traits from *Pisum sativum* (pea) leaves that addresses these challenges through generative artificial intelligence. Our pipeline integrates imaging, detection, segmentation, and synthetic data generation processes. A nail polish impression technique was employed to prepare leaf microscopic images, followed by the application of deep learning networks to identify and segment stomata in these images. By including generative AI-produced synthetic data, our system achieves high segmentation accuracy across species, reducing manual relabeling requirements. This approach enables seamless cross-species model adaptation for many cases, alleviating the annotation bottleneck that often limits machine learning applications in plant biology. Our results demonstrate the pipeline’s effectiveness for automated stomatal trait extraction and highlight generative AI’s transformative potential in advancing stomatal detection methodologies, offering a scalable solution for broad-scale comparative stomatal analysis.

**Supplementary Information:**

The online version contains supplementary material available at 10.1186/s13007-025-01451-z.

## Background

Stomata are microscopic pores on plant surfaces that function as gateways for gas exchange and water regulation [1]. By opening and closing to control airflow and water movement, these structures are fundamental to photosynthesis and temperature regulation in plants. Studies examining stomatal traits, including density, morphology, aperture status and size, provide critical insights into the physiology and performance of plants. Stomatal density and area serve as indicators for estimating photosynthetic capacity and transpiration rates. While increased stomatal density typically enhances $CO_{2}$ uptake, it simultaneously elevates water loss, creating trade-offs that affect plant water-use efficiency under different environmental conditions [2]. Similarly, stomatal area, determined by guard cell dimensions, influences gas diffusion rates, with larger pores enabling faster exchange while potentially compromising precise water loss control [3]. These morphological features represent key markers of plant environmental adaptation, as demonstrated by documented shifts in stomatal characteristics in response to elevated $CO_{2}$ concentrations and drought conditions [4]. Advancing our understanding of these parameters will significantly improve our capacity to predict plant responses to climate change and enhance agricultural productivity through informed crop optimization strategies.

Breakthroughs in machine learning-based object detection and segmentation have provided new opportunities for automated stomatal trait analysis [5–8]. Deep learning approaches enable efficient, automated extraction of morphological characteristics, unlocking research possibilities and experimental designs that were previously prohibitively complex or expensive. Multiple studies have successfully extracted stomatal traits from plant imagery using diverse methodologies. Pathoumthong et al. developed a handheld system for stomatal analysis [9], while Liang et al. similarly employed portable imaging technology for stomata detection [5]. For segmentation applications, Gibbs et al. implemented a U-Net architecture to identify stomatal substructures [10]. Extending beyond static image analysis, Sun et al. created a video-based system capable of real-time tracking and monitoring of stomatal dynamics [11]. These developments collectively demonstrate the growing sophistication of automated approaches to stomatal research, transitioning from manual measurement techniques to intelligent, scalable analytical frameworks.

While numerous implementations have produced effective stomatal trait analysis tools, existing machine-learning based detection methods face significant limitations. These approaches consistently require laborious manual annotation for model training. Additionally, current models exhibit poor cross-domain transferability, and applying trained models to datasets with different characteristics typically necessitates comprehensive relabeling, because existing annotations are insufficient. Dataset variations may arise from different plant species, imaging protocols, or microscopy techniques, each introducing distinct visual characteristics that compromise model performance. To address these challenges, we propose leveraging generative AI models for cross-domain data translation.

Generative AI has become a transformative force in computer vision, particularly for creating synthetic training data in instance segmentation applications. Through generative models such as Generative Adversarial Networks (GANs), researchers can produce high-quality, diverse training masks that enhance segmentation algorithm robustness. These synthetic datasets enable models to learn from expanded example sets, improving generalization capabilities on real-world data. Pioneering work by Barth et al. [12] demonstrated GAN-based generation of realistic training masks that replicate complex patterns and textures characteristic of natural scenes. Numerous studies have subsequently validated generative AI applications in training dataset preparation [13–15], highlighting the potential of generative approaches to transform computer vision dataset development. Our approach employs generative networks to perform style transfer on existing dataset images to match the visual characteristics of newly acquired data. This strategy significantly reduces or even eliminates labor-intensive manual re-annotation requirements under certain circumstances.

This research presents a comprehensive machine-learning powered framework that integrates leaf imaging, stomatal detection, trait quantification, and synthetic data generation into a unified analytical system. This study demonstrates how generative AI models can facilitate dataset translation between plant species, enabling effective cross-domain knowledge transfer. By combining synthetic data generation with basic transfer learning methodologies, our pipeline achieves substantial reductions in manual annotation requirements particularly when the source and target datasets share similar morphological characteristics. Our findings validate the effectiveness of generative AI in creating synthetic training data in stomatal analysis, providing a scalable approach for automated trait extraction across diverse plant species while minimizing the need for extensive species-specific labeling.

## Material and methods

### Plant growth and imaging

**Fig. 1:**
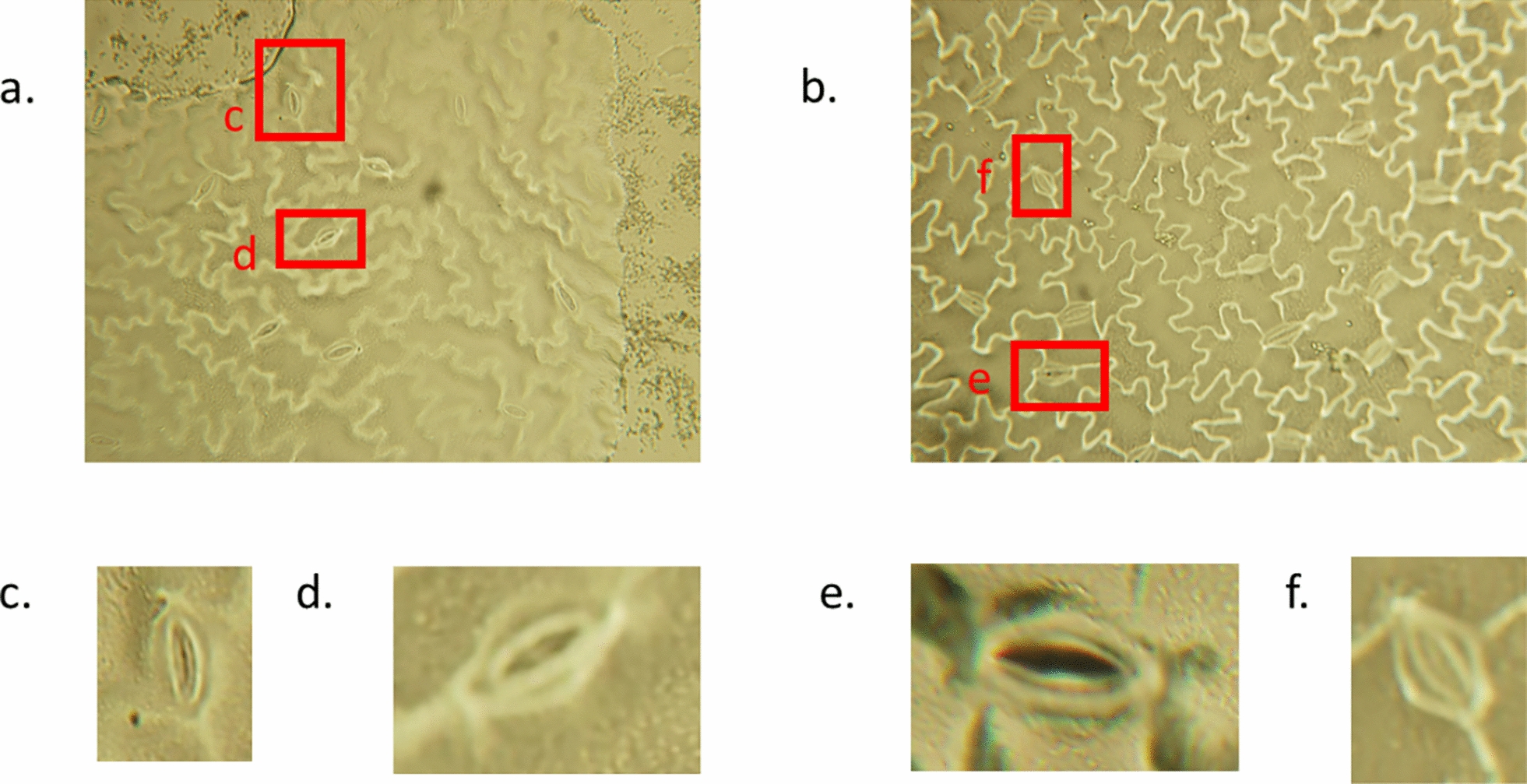
*Pisum sativum* (pea) leaf print images taken using a Leitz Diaplan microscope with an Olympus DP70 digital camera. **a**,** b** Original images from the microscope camera; image size is 4080x3072 pixels with a resolution of around 1 $\upmu$m per pixel. **c**,** d** and **e**,** f** are zoomed in images from panels (**a**) and (**a**) respectively; image size is around 200x300 pixels

Pea plants (*CDC Amarillo*) were grown in a controlled environment chamber under the following conditions: light intensity of $\upmu \textrm{mol}\, \textrm{m}^{-2} \textrm{s}^{-1}$, $60\%$ relative humidity, and 16-hour light/8-hour dark photoperiod with corresponding temperatures of 22 °C and 20 °C, respectively. Fully expanded true leaves were harvested near the basal region of the stem from 15-day-old pea plants, and the abaxial surface was immediately coated with a thin layer of clear nail polish [16]. The coated leaves were pressed between two microscope slides to create an impression, after which the leaf tissue was carefully peeled away, leaving an epidermal imprint on the slide. A total of 81 lines were grown, with each line having three replicates, and three plants were grown per replicate. One plant per replicate was used for epidermal impressions ($n = 243$). Light micrographs were acquired within one hour of preparation using a Leitz DIAPLAN microscope (Fig. S1 in Supplementary Material) with an Olympus DP70 camera and 40x objective, with an average of ten images captured per epidermal peel (Fig. 1). Default camera settings were used with no pre-processing white-balancing algorithm.

### Stomatal trait detection network

A two-stage approach was adopted for stomatal trait detection: initial localization through bounding box extraction followed by semantic segmentation of individual stomata. Two variants of You Only Look Once (YOLO) networks [17], YOLO11 and YOLO11-seg, were used for stomatal detection and segmentation respectively. YOLO11 represents the latest iteration in the YOLO family [17], a series of real-time object detection models recognized for their optimal balance of processing speed, detection accuracy, and ease of implementation in computer vision applications. YOLO architectures and their variants have demonstrated widespread success across diverse segmentation applications  [18–20], with proven effectiveness specifically in stomatal detection research  [21, 22].

### Synthetic training labels from generative AI

**Fig. 2:**
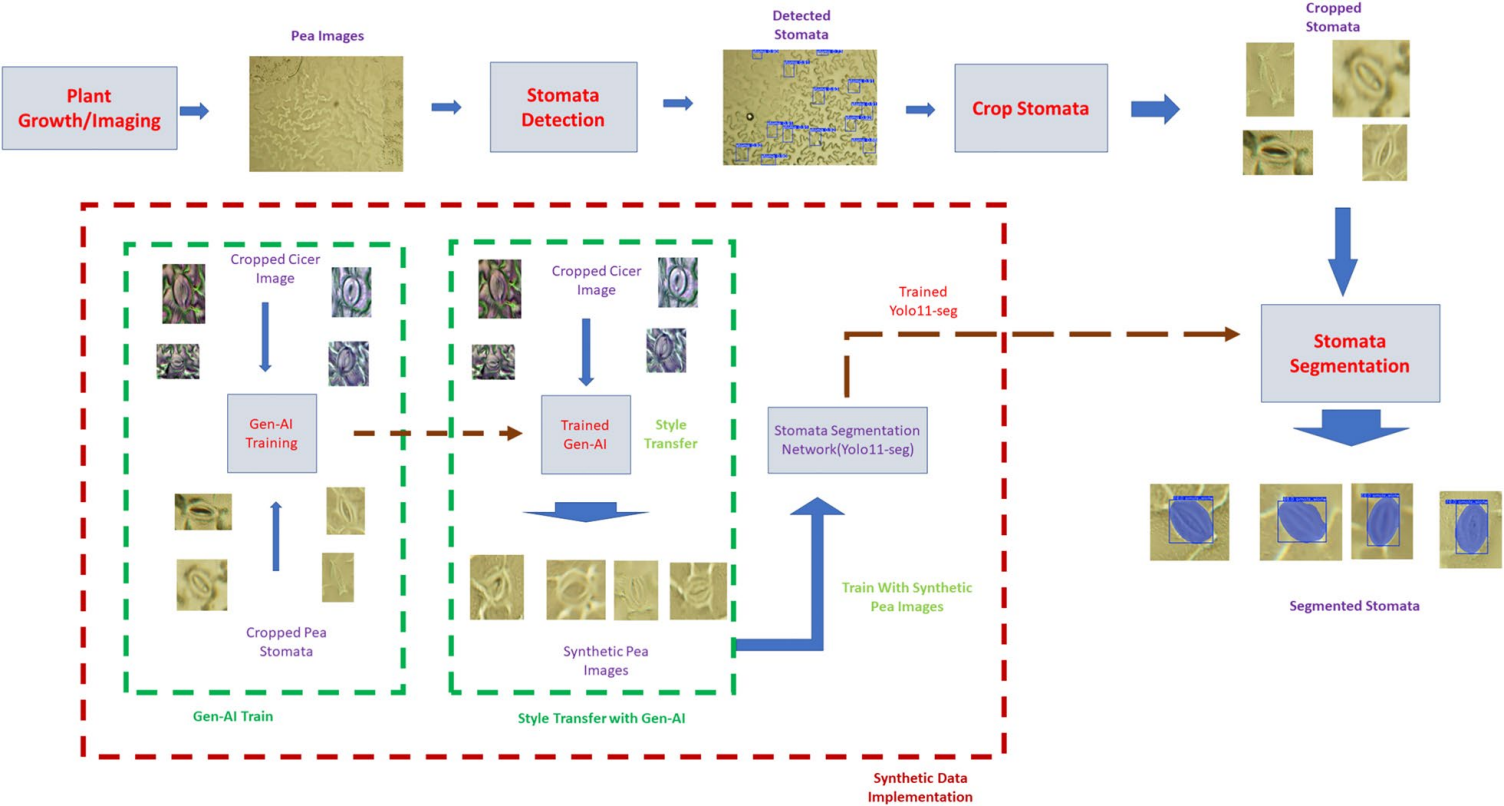
Flowchart of integrating stomatal images with synthetic data. The top panel contains the steps of image processing and stomatal detection. The portion in red-dashed box is the synthetic image generation, the generated synthetic images are used to train the stomatal segmentation network

To use synthetic data, a publicly available dataset with pre-annotated stomatal masks was selected as the reference dataset. Various generative AI models were then employed to ’translate’ the reference dataset images to our pea synthetic images. The resulting pea synthetic images served as the training dataset of the segmentation algorithm. Fig. 2 shows the integration of synthetic data into the stomatal imaging pipeline.

#### Reference dataset

**Fig. 3:**
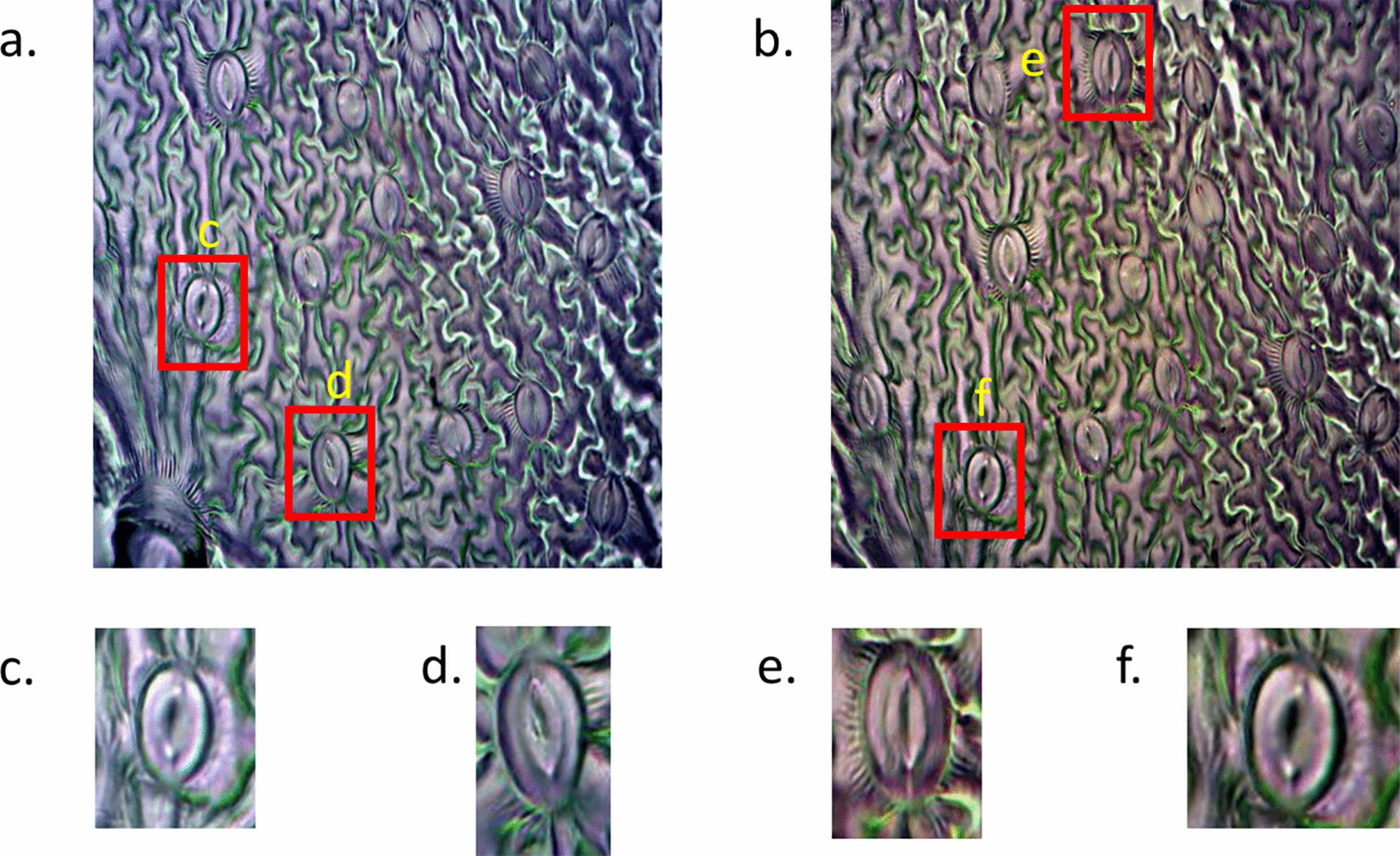
Sample images of the *Cicer arietinum* (chickpea) dataset and the dataset is publicly available at [23]. **a**,** b** Original images from the dataset. **c**,** d** and **e**,** f** are zoomed in images from panels (**a**) and (**b**) respectively

A dataset containing *Cicer arietinum* (chickpea) stomata images, referred to as the *Cicer arietinum* control dataset, was selected as the reference dataset [23]. Sample images from this dataset are shown in Fig. 3. The dataset comprises 766 chickpea images with corresponding stomatal masks. Using these stomatal masks, 7, 164 cropped chickpea stomatal images were extracted. As illustrated in Fig. 3, the chickpea images exhibit substantially different characteristics compared to the pea images (Fig. 1). We subsequently evaluated multiple style-transform generative AI models to convert the cropped chickpea stomatal images into synthetic pea stomatal images. The generated synthetic pea images were then paired with the original chickpea image masks to create training data for our semantic stomatal segmentation network.

#### Cross-validation dataset

**Fig. 4:**
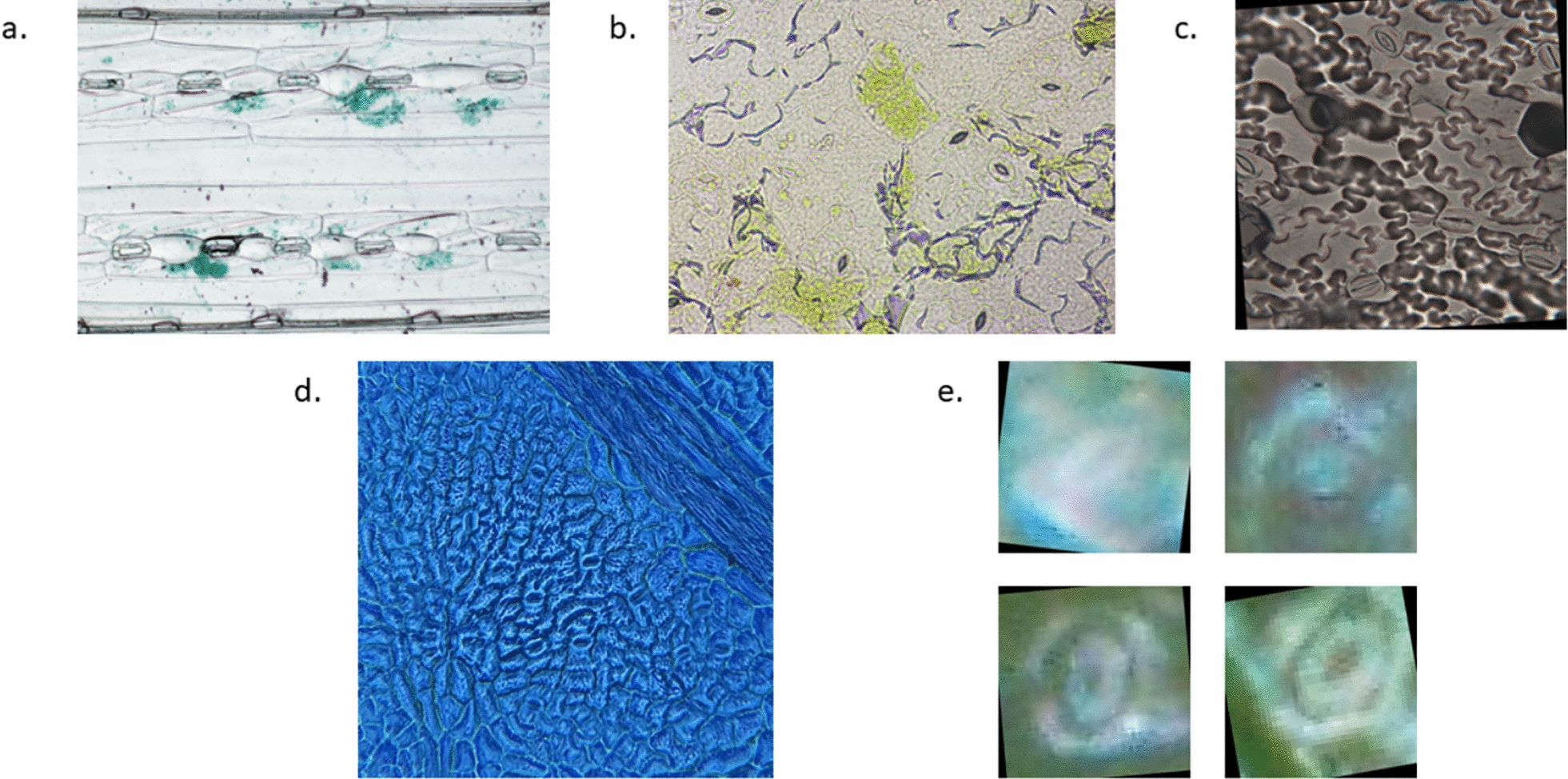
Sample images of the cross-validation datasets, Blurred *Arabidopsis* only contains individual cropped stomatal images, all other species have the original slides from the microscope. **a** A sample image of barley. **b** A sample image of *Arabidopsis*. **c** A sample image of *Chrysanthemum*. **d** A sample image of Norway maple. **e** Several images of Blurred *Arabidopsis*

To assess the effectiveness of the synthetic image generation method, we incorporated several publicly available stomatal segmentation datasets into the cross-validation experiments. Two high-quality datasets from a previous study [24], comprising samples of *Hordeum vulgare* (Barley) and *Arabidopsis thaliana*, were included as benchmarks for reliable stomatal imaging. To represent lower-quality imaging conditions, we further included an *Arabidopsis thaliana* dataset with markedly blurred images [25], hereafter referred to as the Blurred *Arabidopsis* dataset. In addition, to enhance species diversity and capture variation in color characteristics, we incorporated two further datasets: *Chrysanthemum * imprints [26] and *Acer platanoides* (Norway maple) [27]. Representative sample images from each dataset are shown in Fig. 4. For cross-validation experiments, the Norway maple and *Arabidopsis* datasets were randomly subsampled so that all datasets contained approximately 1000–1500 individual stomatal images with corresponding segmentation masks. The exact number of samples per dataset is summarized in Table 4. Among all datasets, only the Blurred *Arabidopsis* dataset consisted solely of pre-extracted individual stomatal images and masks. In contrast, the other datasets included large images containing multiple stomata, each annotated with its own segmentation mask (Fig. 4). For these multi-stomata datasets, bounding boxes could be generated, enabling evaluation of stomatal detection performance across different species and color conditions.

### Generative network

All the selected generative networks require only unpaired data for training, therefore the images did not need any human labeling. The generative network served as a ’style’ transformer that translates images of chickpea ’style’ to pea ’style’, examples of each ’style’ could be found in Figs. 1 and  3. The ’style’ of a dataset captures the general characteristics of the dataset, such as species, size, color tone, and lighting conditions. The generative networks selected were CycleGAN, SpCycleGAN, and UVCGAN.

*CycleGAN*

The Generative Adversarial Network (GAN) [28] were among the first network architectures that popularized the field of synthetic data generation. Cycle-Consistent Adversarial Networks (CycleGAN) [29] is an advanced type of GAN developed to address the challenge of image-to-image translation tasks without requiring paired examples. Introduced by Zhu et al. in 2017, CycleGAN leverages two sets of GANs to transform images from one domain to another while preserving key characteristics and content. It employs a cycle-consistency loss, ensuring that when an image is translated to another domain and back, it remains similar to the original. This innovative approach enables CycleGAN to perform tasks such as transforming summer photos into winter scenes or converting horses into zebras without the need for corresponding samples. CycleGAN has gained popularity for its effectiveness in style transfer and domain adaptation, representing a significant advancement in unsupervised learning techniques for image processing.

*SpCycleGAN*

Spatially Constrained Cycle-Consistent Adversarial Networks (SpCycleGAN) [30] is a variation of CycleGAN in which an additional structure emphasizing spatial consistency is incorporated into the generator to reduce the loss. SpCycleGAN could substantially improve output quality when the input and output images shared structural relationships.

*UVCGAN*

Unsupervised Visual Concept Learning Generative Adversarial Network (UVCGAN) is a novel approach in the realm of generative models that focuses on unsupervised learning of visual concepts [31]. Like CycleGAN, UVCGAN leverages an encoder-decoder structure that autonomously transforms image styles. The encoder and decoder structure of UVCGAN is a Vision Transformer (ViT) [32] based generator called UNet-ViT generator that integrates U-Net [33] and a pixel-wise vision transformer. UVCGAN then adopts a CycleGAN-like framework to enforce cycle-consistency of the ViT encoder and decoder. The design of UVCGAN enables the generation of diverse, high-quality images that align with learned visual concepts, making it a significant contribution to the generative modeling landscape.

### Experiment settings

#### Stomata detection

To validate the performance of YOLO11 in stomatal detection, 100 pea images were manually labeled and split into an 80/20 training/testing split. Training was conducted over 200 epochs with a batch size of 16 and learning rate of 0.01, while utilizing the Ultralytics implementation [34]. For evaluation metrics, sample size, pretraining status, precision, and bounding box mAP at $50\%$ thresholds [35] were reported. To further enhance the model accuracy through transfer learning, YOLO11 was first pre-trained on the combined chickpea and pea dataset, followed by fine-tuning in which the initial two layers were frozen and the remaining parameters were updated using pea images with training continued for an additional 200 epochs at a reduced learning rate. Complete training specifications are provided in the Supplemental Materials. The resulting trained model was applied to extract cropped pea stomatal regions, producing approximately 27, 097 individual stomata images for subsequent analysis from the 2432 original images.

#### Stomatal segmentation

For synthetic dataset training, the reference dataset consisted of 7,164 cropped chickpea stomatal images, which were used to train against the 27,097 cropped pea stomatal images from previous steps using various generative AI networks. The CycleGAN employed a ResNet9 backbone generator, a batch size of 16, a learning rate of 0.0002, and was trained for 140 epochs. L1 loss was applied to adversarial, cycle-consistency, and identity loss components. Similarly, SpCycleGAN applied a ResNet9 backbone generator, batch size of 16, learning rate of 0.0002, and was trained for 140 epochs. L1 loss was applied to adversarial, cycle-consistency, and identity losses, while a mean squared error (MSE) loss function was applied for the spatial-consistency constraint. For UVCGAN, the Vision Transformer–based generator was pretrained for 550 epochs with a batch size of 16, after which the entire network was trained for an additional 150 epochs with a batch size of 8 to enforce cycle-consistency. Detailed training parameters are provided in the Supplemental Materials. The resulting generative networks translated the 7, 164 chickpea stomatal images with segmentation masks into a synthetic dataset comprising 7,164 synthetic pea stomatal images with corresponding segmentation masks, as illustrated in [29].

The quality of the synthetic images was assessed using the Fréchet Inception Distance (FID) score [36], calculated with an InceptionV3 network [37] pretrained on the ImageNet dataset. The quality of the synthetic images was further evaluated based on the performance of the segmentation network trained with synthetic data. YOLO11-seg was employed for stomatal segmentation on synthetic images generated by various generative AI networks. The network was trained with an input image size of 256, a learning rate of 0.01, and 200 epochs. Detailed training parameters are provided in Supplemental Materials. Mask mAP at the $50\%$ threshold and F1 score were reported to evaluate segmentation performance. Additionally, basic transfer learning was applied: the segmentation network was pretrained on a combined dataset of real chickpea and synthetic pea images, and then fine-tuned on synthetic pea stomatal images with a reduced learning rate. A manually segmented test dataset containing 197 pea stomatal images was used for all segmentation metrics. In addition, a manually segmented reference training set of 191 images was created to compare performance against the synthetic data–trained segmentation results. Our aim was for the segmentation network trained on synthetic data to achieve results comparable to networks trained on manually segmented data.

#### Stomata detection and segmentation cross validation

To validate YOLO11’s stomatal detection capabilities across multiple plant species and imaging conditions, the same training approach developed for pea images was applied. From each cross-validation dataset, 100 images with their corresponding masks were randomly selected and allocated to training (80 images) and testing (20 images) sets. All models were trained for 200 epochs using a batch size of 16 and learning rate of 0.01, implemented via the Ultralytics framework [34]. Model performance was assessed using precision and mean Average Precision (mAP) metrics at the 50% confidence threshold threshold  [35].

For segmentation validation, six distinct datasets were utilized: five cross-validation sets (*Arabidopsis*, Blurred *Arabidopsis*, Barley, *Chrysanthemum*, and Norway maple) and one reference dataset created by subsampling 1500 pea images. SpCycleGAN was employed to generate synthetic data for cross-species segmentation validation across varying visual conditions. Individual SpCycleGAN models were trained using the chickpea reference dataset as the source domain for each target cross-validation dataset. Each SpCycleGAN model employed a ResNet9 backbone generator and was trained with specific parameters: batch size of 16, learning rate of 0.0002, and 140 epochs. This process generated six unique models, each capable of transforming the 7, 164 cropped chickpea stomatal images into 7, 164 corresponding synthetic images that matched the visual characteristics of the target species. The resulting synthetic images were then used to train individual YOLO11-seg segmentation networks. These networks used consistent parameters across all species: 256-pixel input resolution, 200 training epochs, and 0.01 learning rate. The loss function was also the same as pea dataset. Performance evaluation was conducted using test sets of 197 randomly selected images from each corresponding cross-validation dataset. All network configuration details are available in the Supplemental Materials.

**Fig. 5:**
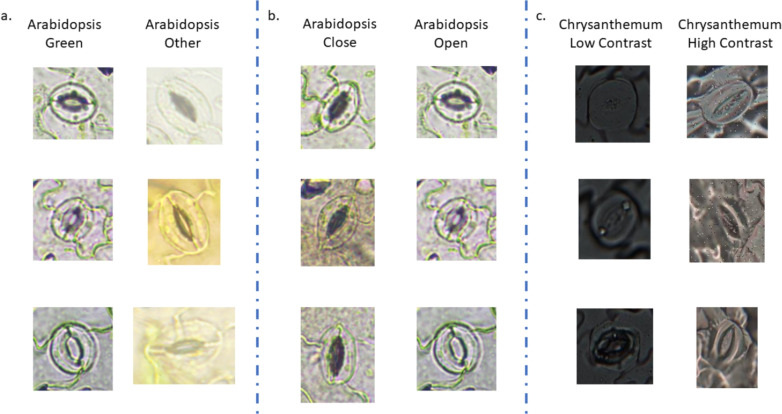
Sample images of stomata with different characteristics. **a** Green *Arabidopsis* and other colored *Arabidopsis* images **b** Green *Arabidopsis* images with opened and closed stomata **c** *Chrysanthemum* images with high contrast/illumination and low contrast/illumination

To evaluate the method’s robustness under different conditions, segmentation performance was analyzed across various stomatal characteristics. Significant color variation was observed within the Arabidopsis dataset, with some images appearing considerably greener than others (Fig. 5). To investigate this variation systematically, segmentation results were computed separately for 100 green Arabidopsis stomata and 100 standard Arabidopsis stomata. Stomatal state effects were further analyzed by randomly sampling 50 open and 50 closed stomata from the green Arabidopsis subset. Additionally, the impact of illumination conditions was assessed by selecting 50 low-contrast and 50 high-contrast Chrysanthemum stomatal images for comparative performance analysis.

#### Dataset and training resources

The experimental framework incorporated several interconnected datasets with specific allocation purposes. The primary pea dataset contained 2,432 images, from which 100 were manually annotated with bounding boxes for stomatal detection (distributed as 80 training and 20 testing images). Stomatal segmentation analysis yielded 27,097 individual pea stomatal images extracted from the original dataset. The chickpea reference dataset comprised 766 source images, generating 7,164 cropped individual stomatal images. Table 4 provides detailed counts of extracted stomatal images across all cross-validation datasets. Synthetic image generation incorporated all individual stomatal images from both pea and cross-validation datasets during training against respective validation sets. A total of 197 pea images were manually annotated to serve as the primary test dataset for segmentation performance evaluation. An additional 191 pea images were labeled to create the training set for the reference segmentation network. Similarly, each cross-validation dataset contributed 197 images with corresponding segmentation masks for testing purposes. All generative AI network training was conducted using four NVIDIA A100 GPUs from the High-Performance Computing facility, equipped with 128 AMD EPYC 7532 32-Core CPU cores and 1TB shared memory allocated to the GPU partition. All YOLO-based detection and segmentation tasks were executed using two Tesla V100 GPUs from the same computational facility.

## Results

**Fig. 6:**
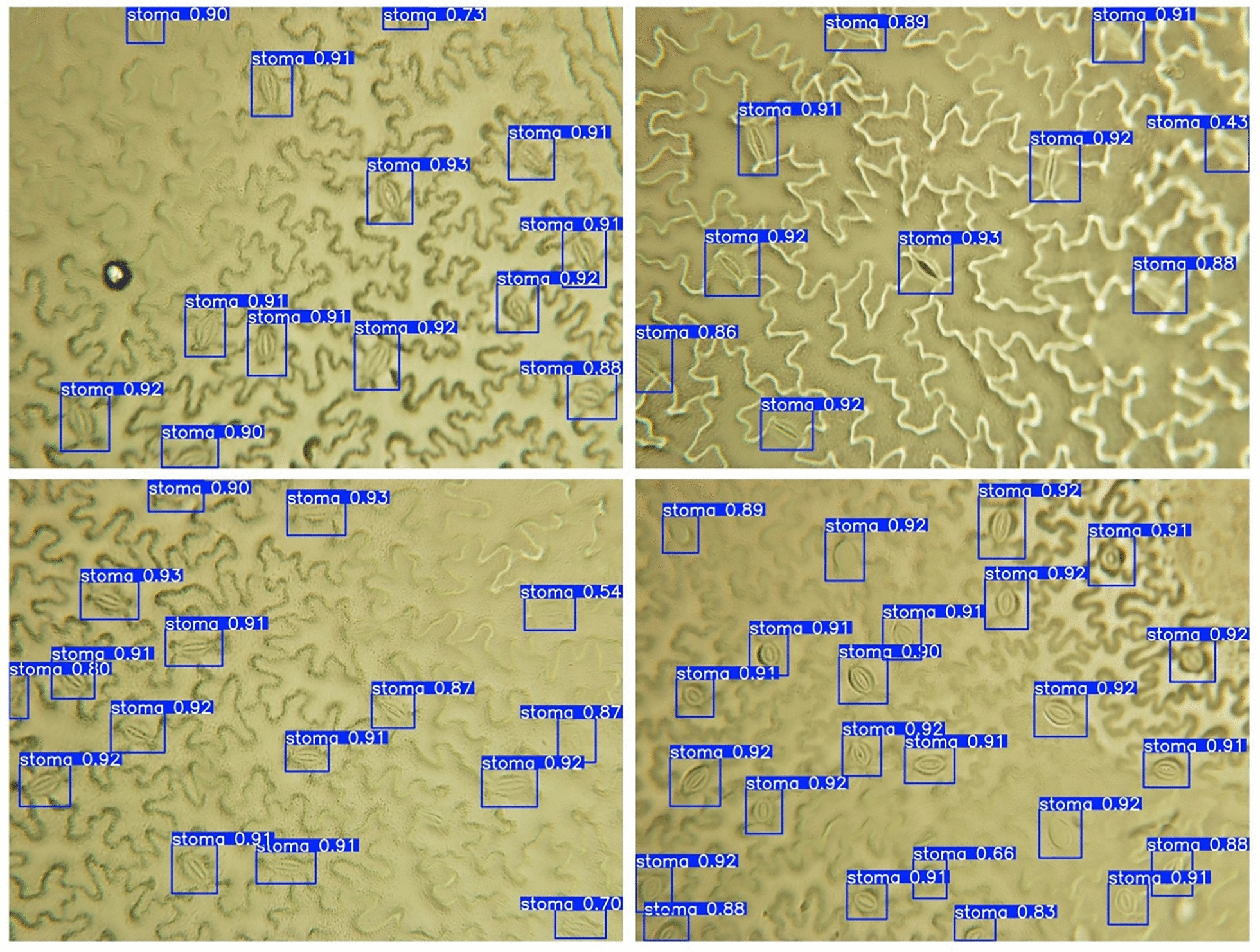
Detection results for pea stomata detection using YOLO11, the detected stomata are in the blue bounding boxes

### Stomata detection

The stomata detection network YOLO11 was trained to identify and localize individual stomatal structures within microscopic leaf surface images, generating precise bounding boxes around each detected stoma. To further enhance detection performance, transfer learning techniques were systematically applied by leveraging pre-trained weights from models trained on broader botanical datasets. Fig. 6 presents representative examples of detected pea bounding boxes overlaid on test dataset images, demonstrating the model’s capability to accurately identify stomatal structures under varying imaging conditions. Quantitative performance evaluation of pea images was conducted using standard object detection metrics, with results comprehensively presented in Table 1.

**Table 1:** Stomatal detection results

	Total sample size	Pretrain status	Precision	Box mAP@$50\%$
Pea	2432	No	0.926	0.935
Pea (transfer learning)	2432	Yes	0.953	0.964
*Arabidopsis*	242	No	0.937	0.979
Barley	155	No	0.961	0.991
*Chrysanthemum*	110	No	0.948	0.973
Norway maple	133	No	0.864	0.835

In Table 1, the baseline YOLO11 model for pea images achieved a precision of 0.925 and an mAP@$50\%$ score of 0.935. Transfer learning was able to slightly improve both metrics, with the precision increasing to 0.953 and the mAP@$50\%$ score improved to 0.964. This improvement can be attributed to the model’s enhanced ability to generalize stomatal features across different species and imaging conditions through the incorporation of additional data.

The visualization reveals that YOLO11 exhibits robust performance in distinguishing stomata from surrounding epidermal cells, capturing most visible stomatal apertures even in challenging scenarios with varying illumination, focus conditions, and tissue density. The detected bounding boxes consistently encompass the complete stomatal complex, including both guard cells and the central pore, which is essential for subsequent morphological analysis. Overall, the detection model demonstrates reasonable accuracy in stomatal counting tasks, which is critical for applications requiring precise stomatal density measurements.

At the image peripheries, stomatal structures are only partially visible within the field of view, leading to unreliable size estimations and potentially biased statistical analyses. To address these edge-related detection issues, a border exclusion zone of 150 pixels was established around each image perimeter, approximately corresponding to the typical length of a complete stomatal complex as determined from manual measurements of fully visible structures. Detection filtering was performed using an overlap threshold criterion, where any bounding boxes exhibiting more than $80\%$ spatial overlap with the defined border region were automatically discarded from the final results. This buffer distance ensures that only stomata with complete morphological features are retained for analysis. These extracted images served as the foundation for subsequent processing steps, including morphometric analysis, synthetic data generation, and comparative studies. The standardized extraction process ensured that all retained stomata possessed sufficient morphological details for reliable automated analysis.

The stomatal detection results for cross-validation datasets are presented in Table 1. It is important to note that the results for all datasets are obtained by training on 80 images and testing on 20 images while total sample size only record the total number of images in each dataset. All the tested datasets resulted in similar precision and mAP@$50\%$ as the reference dataset, indicating robust performance across varying plant species and imaging conditions. Due to the small sample sizes of the cross-validation datasets, stomata located near the image peripheries were not removed.

**Fig. 7:**
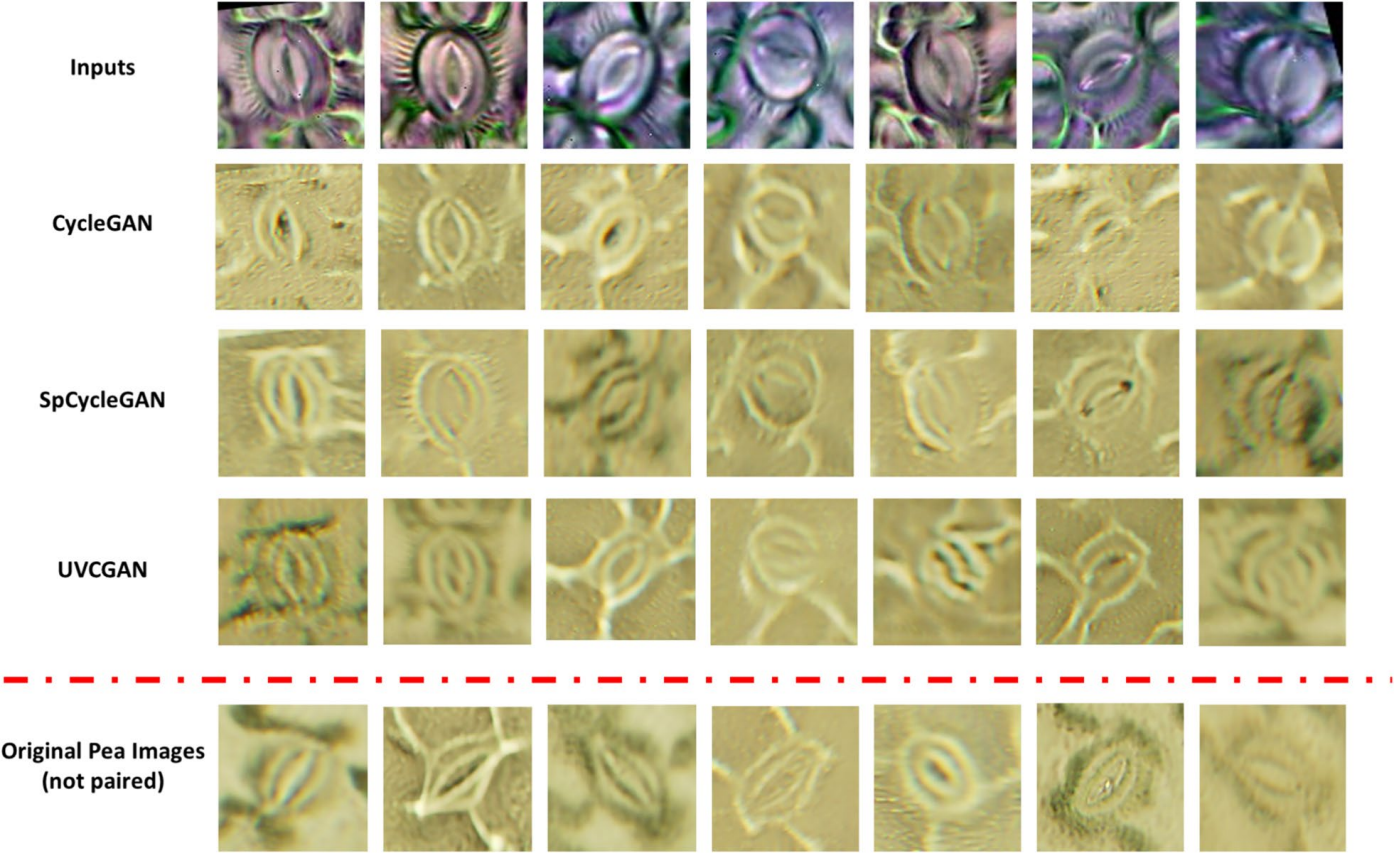
Synthetic pea images translated from chickpea images using different generative networks. Input row contains the original cropped chickpea images, followed by their corresponding synthetic pea images from each generative AI method. Several sample reference pea images are also included, note that they are not paired to the images from the input row

### Synthetic image quality study

CycleGAN, UVCGAN, and SpCycleGAN were trained to perform domain translation from chickpea stomatal images to the pea stomatal image domain. Fig. 7 displays representative examples of the generated synthetic pea stomatal images derived from their corresponding chickpea source images. The inputs are the original chickpea stomatal images, while the subsequent rows showcase the corresponding synthetic pea stomata images produced by various generative architectures. There are also several reference original pea images at the bottom for comparison, the reference images are not paired with the input. Each method demonstrates similar characteristics in terms of texture synthesis, structural preservation, and visual fidelity.

A qualitative assessment was conducted through detailed visual inspection by plant scientists with expertise in stomatal morphology. The evaluation focused on key anatomical features, including guard cell structure, stomatal pore definition, subsidiary cell arrangement, and overall tissue organization. According to these expert evaluations, all generative networks successfully produced high-quality synthetic images that maintained biological plausibility and captured the essential morphological features characteristic of pea stomata. The synthesized images exhibited appropriate cellular textures, realistic stomatal aperture shapes, and convincing integration with surrounding epidermal tissue.

**Table 2:** FID for different generative networks

	CycleGAN	SpCycleGAN	UVCGAN
FID	28.22	24.16	36.41

Additionally, quantitative evaluation was performed using the Fréchet Inception Distance (FID), a widely adopted metric for assessing the quality and diversity of generated images. Table 2 presents the FID scores for all evaluated generative models. Among the tested architectures, SpCycleGAN demonstrated superior performance with the lowest FID score of 24.16, indicating the closest statistical similarity between generated and real pea stomatal images. CycleGAN achieved a competitive FID score of 28.22, followed by UVCGAN at 36.41. These scores represent substantial improvement over the baseline domain gap, as evidenced by the FID score of 221.7 between the original chickpea and original pea datasets, highlighting the significant differences between the two species.

**Fig. 8:**
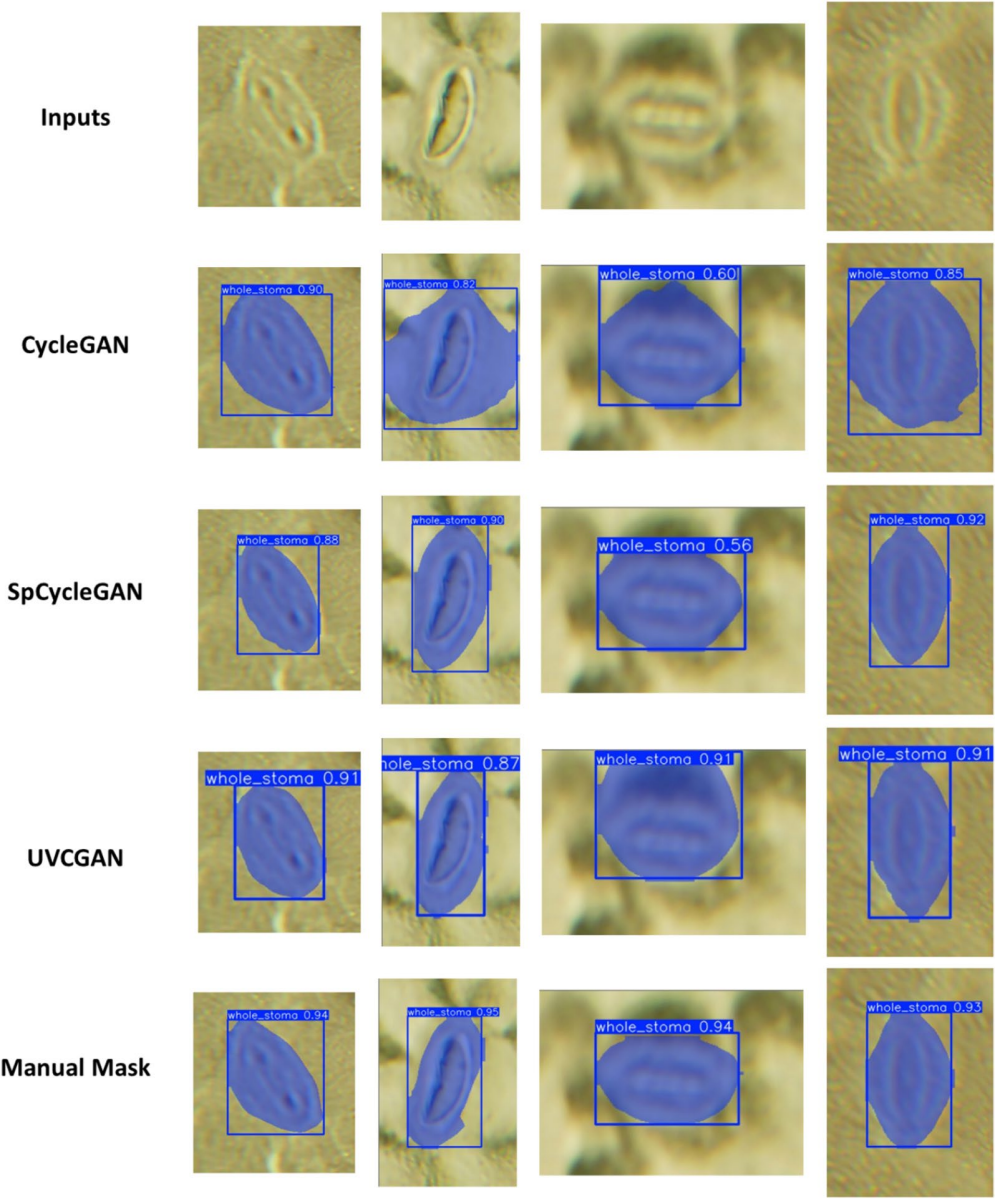
Segmentation results of YOLO11-seg trained with datasets from different sources. Input row contains original cropped pea images, followed by YOLO11-seg results trained by each of the synthetic data generation methods. Manual Masks are images from the reference YOLO11-seg network trained by manually labeled data

### Segmentation using synthetic dataset

Several YOLO11-seg networks were trained on synthetic datasets generated from various generative AI networks. Fig. 8 demonstrates sample images of segmentation results: the inputs are original pea images, followed by the YOLO11-seg segmentation result trained with synthetic data from the three selected generative AI methods (CycleGAN, SpCycleGAN, UVCGAN). In addition, the figure also contains results of a reference YOLO11-seg segmentation network that was trained with manually segmented data. From the images above, masks from all the networks seem to be able to encompass generalized stomatal features including guard cells and the central pores. Among all three generative AI methods, network trained with SpCycleGAN synthetic data appears to have the closest resemblance to the reference network, where boundaries of the stomatal guard cells are more well defined than others.

**Table 3:** YOLO11-seg segmentation results trained with synthetic pea data

	Mask mAP@$50\%$		F1 Score	
Network	No-pretrain	Pretrain	No-pretrain	Pretrain
CycleGAN	0.863	0.849	0.839	0.823
SpCycleGAN	0.933	0.902	0.896	0.865
UVCGAN	0.877	0.855	0.801	0.813
Ref-Manual	0.967	0.932	0.954	0.941

Our observation can be further validated by the empirical evaluation metric data. The mAP@$50\%$ and F1 scores of each network are presented in Table 3. There is also an entry named Ref-Manual which indicates the reference network trained by manually segmented data. SpCycleGAN proved to be the most effective generative network and the segmentation networks trained with SpCycleGAN-synthetic data achieving the F1 Score of 0.896 and mask mAP@$50\%$ of 0.933, the highest compared to other synthetic data methods. The results from SpCycleGAN closely resemble those of the manually trained reference network, which had an F1 score of 0.954 and mask mAP@$50\%$ of 0.967, respectively. Nevertheless, unlike bounding box detection, incorporating transfer learning seems to have minimal impact on mask accuracy. Overall synthetic data training on YOLO11-seg demonstrates reasonable accuracy in stomata detection tasks with no manually labeled data required, which is critical for applications requiring precise stomatal area measurements. These metrics establish a solid foundation for automated stomata analysis.

**Fig. 9:**
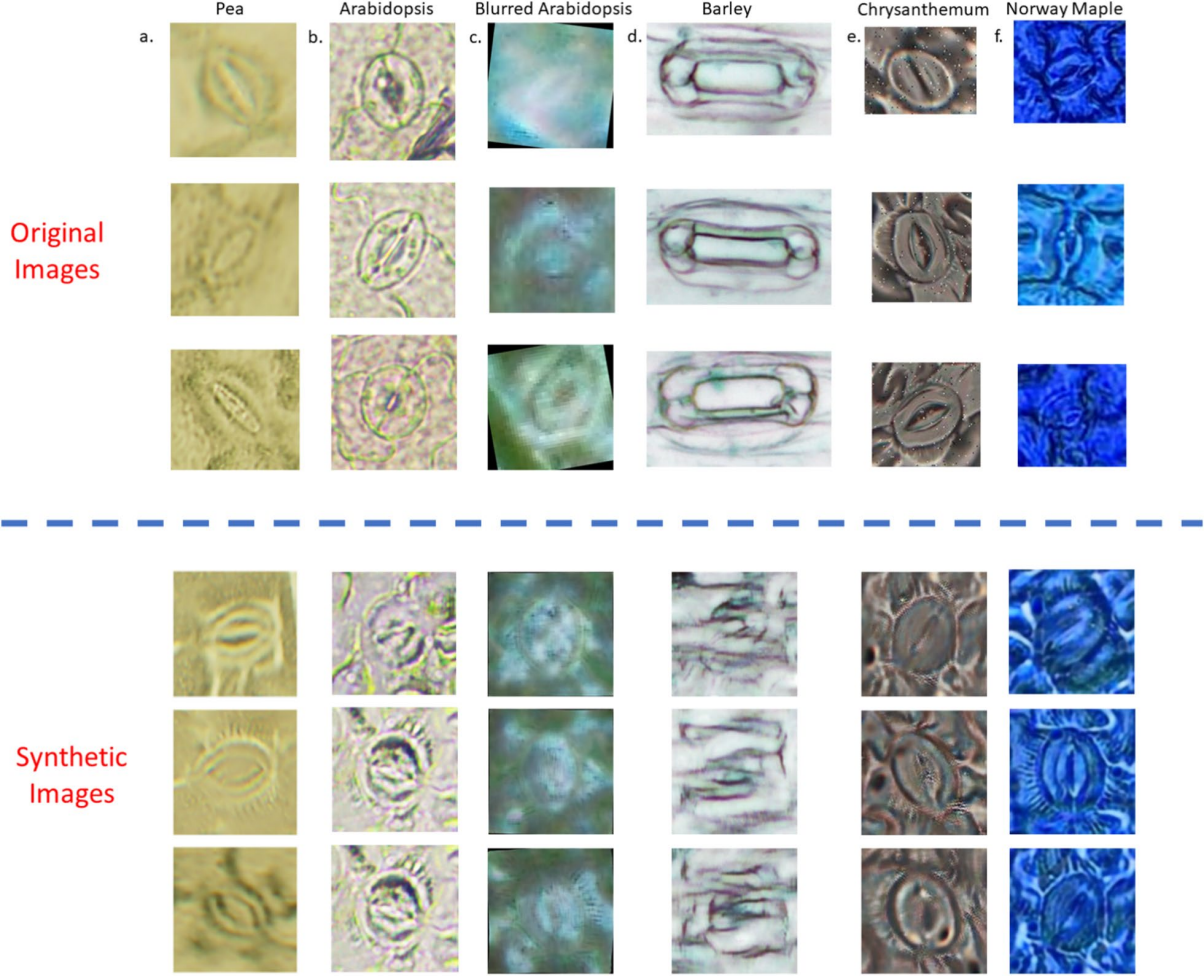
Sample Synthetic images results trained with the cross-validation datasets using SpCycleGAN. Upper portion are original images and lower portion are synthetic images. **a** Sampled 1500 Pea images **b** Sample *Arabidopsis* images **c** Sample blurred *Arabidopsis* images **d** Sample Barley images **e** Sample *Chrysanthemum* images **f** Sample Norway maple images

**Table 4:** Cross-validation stomatal segmentation results

	Sample size	Mask mAP@$50\%$
Pea original	27,097	0.933
Pea 1500	1500	0.695
*Arabidopsis*	1536	0.943
Blurred *Arabidopsis*	1138	0.647
Barley	1703	0.018
*Chrysanthemum*	746	0.878
Norway maple	1500	0.750

### Synthetic data segmentation over different species

We performed synthetic data segmentation analysis using images generated from six cross-validation datasets based on chickpea source images (Fig. 9). From left to right, the datasets include: subsampled pea images (Pea1500), *Arabidopsis*, Blurred *Arabidopsis*, Barley, *Chrysanthemum*, and Norway maple. Table 4 summarizes the dataset sizes along with the corresponding mAP@$50\%$ scores obtained using YOLO11-seg models. The Pea1500 reference dataset achieved an mAP@$50\%$ of 0.695. For all species except barley, the synthetic images were of sufficient quality and produced mAP@$50\%$ values comparable to or exceeding the reference. In contrast, barley performed poorly, with an mAP@$50\%$ of only 0.018, substantially lower than the other species.

Table 5 presents segmentation performance across different stomatal categories, measured by mask mAP@$50\%$. For *Arabidopsis*, stomata labeled as ’green’ reached an mAP@$50\%$ of 0.946, significantly higher than the ’other’ stomata category (0.740). Within the ’green’ group, open stomata achieved an mAP@$50\%$ of 0.974, whereas closed stomata reached 0.919. Additionally, *Chrysanthemum* images with high visual contrast yielded an mAP@$50\%$ of 0.931, outperforming those with low contrast.

**Table 5:** Segmentation results for stomata with different characteristics

	Sample size	Test sample size	Mask mAP@$50\%$
*Arabidopsis* Green	1254	100	0.953
*Arabidopsis* Other	282	100	0.706
*Arabidopsis* Green Open	1082	50	0.919
*Arabidopsis* Green Close	172	50	0.974
*Chrysanthemum* High Contrast	626	50	0.931
*Chrysanthemum* Low Contrast	120	50	0.510

## Discussion and conclusion

### Stomatal traits extraction networks

YOLO11, a variant of the YOLO family, was chosen to be the backbone of our two-step (detection and segmentation) stomatal feature extraction framework. Beyond established example usages in leaf feature detection, YOLO also has a robust ecosystem of available implementations, ensuring computational efficiency and facilitating straightforward deployment across various computing platforms, including resource-constrained mobile devices. The implementation of the two-step strategy could drastically reduce problem complexity and annotation requirements compared with single-stage methods [38]. The detection phase required only bounding box coordinates rather than pixel-level masks, while the segmentation phase operated on cropped stomatal regions, significantly reducing the training data volume needed relative to whole-leaf processing. Additionally, this approach addresses resolution loss during segmentation stage: The cropped stomatal images (approximately $200 \times 300$ pixels) preserve morphological details that would otherwise be compromised during whole-image($3072 \times 4080$ pixels) downsampling, ensuring higher fidelity trait extraction.

### Transfer learning and synthetic data

This study demonstrates that transfer learning could slightly enhance bounding box detection performance, while showing negligible effects on segmentation mask accuracy. We attribute this difference to the distinct nature of the training data used for each task. For stomata detection, transfer learning exposed the network to genuinely novel data, leading to improved detection accuracy. Conversely, the segmentation task utilized both chickpea and synthetic pea images during training. Because the synthetic pea images were generated from the original chickpea dataset, they retained the same underlying feature vectors but were mapped to different statistical distributions. When generative AI transforms data from one domain to another, it preserves the fundamental Gaussian distribution vector of the source data. Consequently, training the segmentation network on both chickpea and synthetic pea images essentially involved two datasets sharing the same distributional vector properties rather than truly diverse data sources. This similarity limited the network’s ability to learn new features that would meaningfully improve segmentation accuracy.

Overall, the pipeline presented here successfully detected and segmented stomata from images. The synthetic images generated (Fig. 7) appear to be of relatively high quality, both empirically and visually. This research has demonstrated the potential of synthetic images in stomatal segmentation. Given similar stomatal morphology and sufficient samples, synthetic training data produced by various generative AI models can achieve accuracy comparable to human-labeled data. By utilizing synthetic images, we were able to reduce, or even eliminate, the need for human labeling in semantic segmentation under many conditions. Our method offers numerous potential applications, including for research in a range of species. Theoretically, there would be no need for human labeling for many plant species, even if the datasets appear very different due to variations in species or imaging techniques. For example, studies monitoring stomatal size across certain species under varying lighting conditions can be conducted with significantly reduced need for human labeling.

### Application on different species and conditions

#### Stomatal detection

Cross-validation experiments across six plant species demonstrate the robustness of our detection framework. All tested species achieved precision rates above 0.93 and mAP@50% scores exceeding 0.695, confirming the general applicability of the YOLO11 architecture for stomatal detection. The consistently high performance across morphologically diverse species from monocot like barley to dicots like *Arabidopsis* indicates the model’s ability to learn generalizable detection features. However, our analysis identifies specific challenges that warrant consideration. Species like Norway maple where their plant stomata sometimes blend into the background show slightly reduced detection accuracy, suggesting the need for more targeted training.

#### Stomatal segmentation

Segmentation performance exhibits greater sensitivity to species-specific morphological features than detection. The variation in mAP@$50\%$ scores across species (ranging from 0.018 for Barley to 0.974 for *Arabidopsis*) reflects the complexity of accurately delineating stomatal boundaries across diverse plant taxa. However, when excluding barley, all species produced relative high-quality synthetic images and achieved mAP scores comparable to or higher than those of the Pea1500 reference dataset, based on results from Fig. 4 and Table 4. Overall, our proposed method demonstrated strong capability in transforming across different species and color conditions, thereby significantly reducing the need for manual ground-truth annotation. Nevertheless, certain conditions may still influence and potentially limit the performance of the method.

#### Stomata morphology and sample size

Morphological similarity seems to be a critical factor in determining the outcome of stomatal segmentation. Among the tested species, barley exhibited the poorest segmentation performance, while *Arabidopsis* achieved the highest. Given that both datasets were acquired by the same researchers under comparable imaging conditions, the disparity is most likely attributable to plant morphology. The effectiveness of our method appears to depend strongly on morphological similarity between source and target stomata. As the only monocot evaluated, barley possesses square-shaped stomata with distinct internal structures, in contrast to the oval stomata of dicot species. Because the original chickpea masks were oval, the translation into barley retained this oval geometry. Consequently, the segmentation network attempted to detect square-shaped stomata using oval-shaped masks, leading to significant errors. Moreover, SpCycleGAN proved more adept at translating stylistic attributes such as color than at modifying stomatal morphology. As a result, synthetic barley stomata retained morphological artifacts from the oval chickpea stomata, further reducing segmentation accuracy. These results suggest, retaining morphological similarity between the reference and target stomata appears to be the most crucial condition for our pipeline. Tasks like transferring between monocot and dicot plants will be difficult because many monocot plants have more square-shaped stomata compared to the oval-shaped stomata of dicots.

Another factor influencing segmentation performance is sample size. As shown in Table 4, the results between the original pea dataset and the Pea1500 subset demonstrates that the mask mAP@$50\%$ increased substantially, from 0.695 to 0.933, with the introduction of additional samples. Importantly, because our method does not require segmentation masks to generate synthetic images, increasing the number of training samples does not proportionally increase the time required for manual ground-truth annotation. Original unannotated images are required to improve model performance.

#### Image quality and other factors

Low-resolution images are often produced by low-resolution imaging systems, including when imaging is done in the field, or with images taken at low magnifications. To simulate this condition, a blurred *Arabidopsis* dataset was produced. As shown in Table 4, clear *Arabidopsis* images achieved higher mAP@$50\%$ than their blurred counterparts, despite using a similar number of samples. Notably, even with very low-quality images with limited training samples, our network still achieved performance comparable to the reference pea dataset. Performance could be further improved with a larger sample size.

Some of the cross-validation datasets exhibited distinct imaging styles. As shown in Fig. 5, the *Arabidopsis* dataset included stomata images that appeared noticeably greener than the rest, with the ’green’ stomata also distinguishable by their file names. Among the 1537 *Arabidopsis* stomata images, 1256 were classified as ’green’, resulting in synthetic images that predominantly reflected this style. The segmentation results in Table 5 indicate that the mAP@$50\%$ for ’green’ stomata was 0.953, whereas the mAP@$50\%$ for non-green stomata was only 0.742. Because our framework is based on SpCycleGAN structure, the network implicitly assumes that training images represent a single style. As a result, the synthetic network tends to learn and replicate the most dominant style. This observation could be tested by comparing low and high contrast *Chrysanthemum* images: the synthetic images and the segmentation performance were heavily biased towards the high contrast *Chrysanthemum* images, which had 5 times more samples than their counterparts. Overall, from Table 5, sample size of a specific plant ’style’ seems to be the dominant factor of segmentation results. In practice, this limitation could be mitigated by dividing images of different styles into separate groups and training them independently.

The effect of stomatal opening status was also evaluated. For the ’green’ *Arabidopsis* dataset, the mAP@$50\%$ for closed stomata was 0.919, whereas that for open stomata was 0.974. This slight difference in performance is likely attributable to the disparity in sample sizes between open (n = 1,085) and closed (n = 174) stomata within the training set. Although additional samples would be required to form a balanced dataset and help eliminate potential confounding factors, these results suggest that stomatal opening status does not significantly decrease synthetic mask segmentation performance, as both open and closed stomata achieved high accuracy with mAP@$50\%$ values above 0.9.

#### Stomatal segmentation cross validation results

In summary, our pipeline demonstrates strong transferability across datasets. A necessary condition for the pipeline to perform well is that the reference and target datasets share similar stomatal morphology. For example, converting chickpea images into *Arabidopsis* images is relatively straightforward, whereas converting chickpea stomatal images into a monocot species such as barley, where stomatal morphology diverges substantially, is more challenging. Additional factors that may influence the quality of synthetic mask segmentation include sample size, image resolution, and consistency in training image style. These factors are well-recognized challenges in most deep-learning networks [39], but they can be mitigated by increasing the number of training samples or separating images of different styles into distinct training groups. Despite limitations such as blurred images and unbalanced datasets, our cross-validation results generally achieved performance comparable to or exceeding that of the reference dataset. Taken together, these findings suggest that, provided sufficient sample size and morphological similarity, the synthetic image pipeline presented herein can perform robustly across diverse plant species and color conditions.

### Conclusion

This research presents an integrated pipeline encompassing plant growth, light microscopy, stomata detection, and automated segmentation. This automatic stomatal trait extraction pipeline shows promising potential for future studies. A key contribution of this work is the integration of generative AI to create synthetic training datasets from chickpea stomata reference data, successfully reducing or even to some extent eliminating the dependency on manual annotation processes for species with similar stomatal morphology. The demonstrated feasibility of synthetic data generation represents a paradigm shift in plant research workflows, enabling the incorporation of multiple species and datasets without requiring extensive human labeling efforts. This approach alleviates a critical bottleneck in botanical image analysis, where manual annotation has traditionally limited research scope and scalability.

### Future work

As this work establishes the foundational feasibility of synthetic image generation for stomatal segmentation, subsequent research should focus on advancing synthetic data quality through more sophisticated generative architectures and refined training methodologies, including enhanced transfer learning strategies. Image quality could also be enhanced through optimized acquisition protocols, such as specimen staining procedures to improve feature contrast. Our method currently assumes a single consistent style within the training images, thereby requiring separate training for different species and color conditions. Variations of conditional style-transfer approaches, such as a modified inversion-based style-transfer diffusion models [40], could be implemented. Such methods would enable the synthetic network to generate images across multiple styles for a more robust model capable of handling diverse species and lighting conditions. Nevertheless, our exploration of cross-species translation using generative AI shows promise for mask reusability across related species. The challenge of translating between taxonomically distant groups, such as monocot and dicot stomata, highlights the need to develop mask-independent synthetic generation approaches. Addressing this limitation would further expand the applicability and impact of automated stomata analysis across diverse plant taxa.

Supplementary Material Images of the growth chamber are in Figure S1 Machine learning models training parameters can be found in the document S2.

## Additional file

Supplementary file 1

Supplementary file 2

## Data Availability

Most of the data and codes are publicly available. Contact author for any other data requests.
