# Supplementary figures and images for: A stomata imaging and segmentation pipeline incorporating generative AI to reduce dependency on manual groundtruthing
Source: Plant Methods. 2025 Nov 13;21:148. doi: 10.1186/s13007-025-01451-z (PMC12613397; doi:10.1186/s13007-025-01451-z)

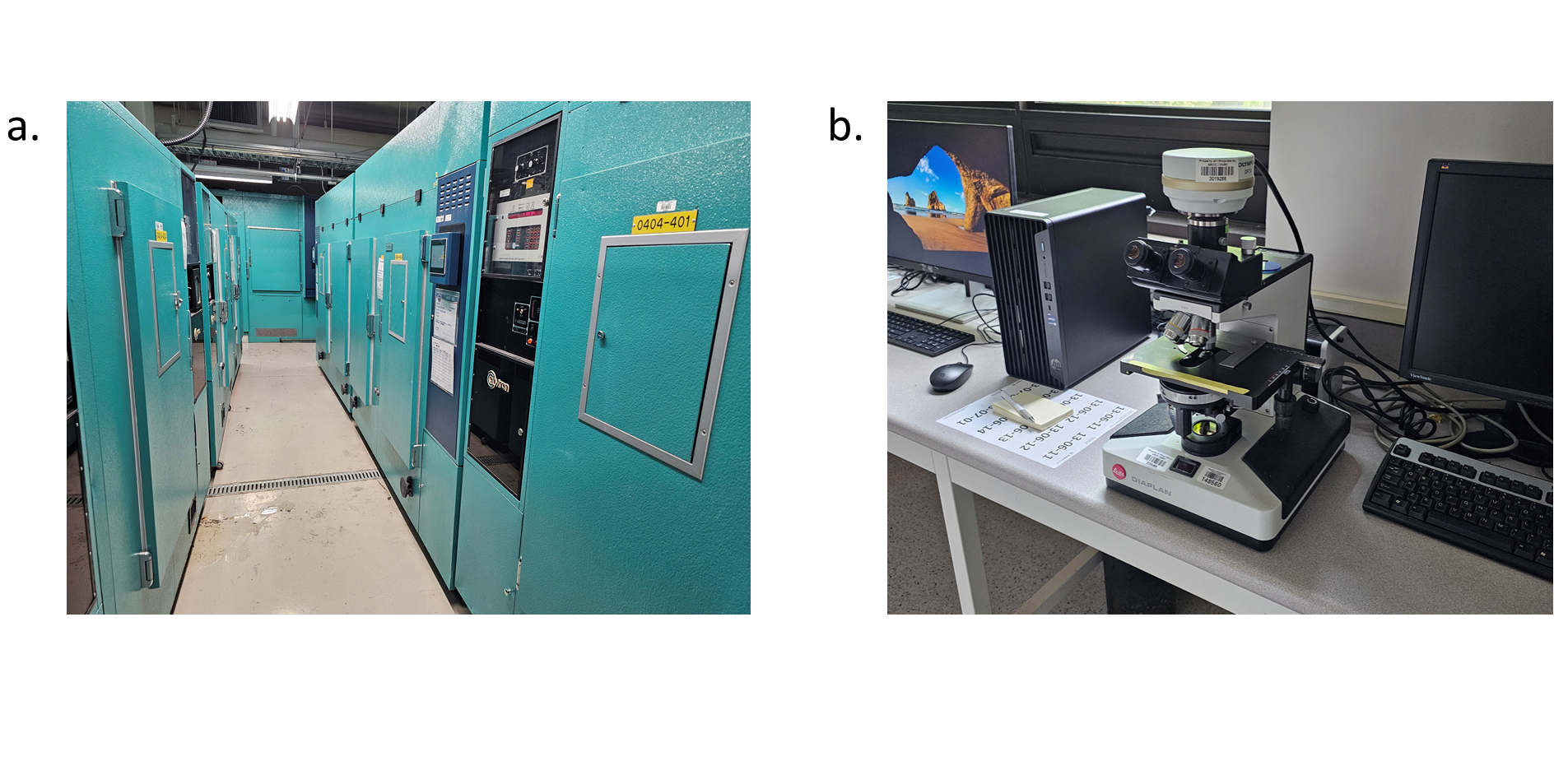

Supplement: Supplementary file 1 [file 13007_2025_1451_MOESM1_ESM.png]
