# Supplementary material for: A stomata imaging and segmentation pipeline incorporating generative AI to reduce dependency on manual groundtruthing
Source: Plant Methods. 2025 Nov 13;21:148. doi: 10.1186/s13007-025-01451-z (PMC12613397; doi:10.1186/s13007-025-01451-z)
Supplement: Supplementary file 2 [file 13007_2025_1451_MOESM2_ESM.docx]

**Stomata Detection:**

Detection:

Yolo Code Location: [Ultralytics YOLO11 - Ultralytics YOLO Docs](https://docs.ultralytics.com/models/yolo11/)

Network : yolo11l, pretrained on ImageNet

Parameters: Epochs:200, learning rate 0.01 , others the same as default on the website

Transfer learning:

Same with detection, except freeze first two layers and reduce lr to 0.002.

Cross Validation:

Same as detection, Epochs:200, learning rate 0.01

**Stomata** **Segmentation:**

Segmentation:

Yolo Code Location: [Ultralytics YOLO11 - Ultralytics YOLO Docs](https://docs.ultralytics.com/models/yolo11/)

Network : yolo11n-seg, pretrained on ImageNet

Changed Parameters: Image size: 256, Epochs:200, mosaic = 0, scale = 0.05, hsv_s = 0.05, hsv_v = 0.05, lr0 = 0.002, explanation is in the online documents

Transfer learning:

Same with Segmentation training, except freeze first two layers and reduce lr to 0.002.

Cross Validation:

Same as segmentation, Image size: 256, Epochs:200, mosaic = 0, scale = 0.05, hsv_s = 0.05, hsv_v = 0.05, lr0 = 0.002, explanation is in the online documents

**Synthetic Data network:**

**CycleGAN:**

Inplementation: [GitHub - junyanz/pytorch-CycleGAN-and-pix2pix: Image-to-Image Translation in PyTorch](https://github.com/junyanz/pytorch-CycleGAN-and-pix2pix)

Most parameters are same as default, changed parameters are: image size 256, no crop / Batch Size 16 / epoch140

**SpCycleGAN:**

Paper: [Three Dimensional Fluorescence Microscopy Image Synthesis and Segmentation](https://openaccess.thecvf.com/content_cvpr_2018_workshops/papers/w44/Fu_Three_Dimensional_Fluorescence_CVPR_2018_paper.pdf)

Most parameters are same as default: image size 256, no crop / Batch Size 16 /epoch140

Cross Validation:

Same as normal SpCycleGAN:

**UVCGAN:**

[GitHub - LS4GAN/uvcgan: Code accompanying UVCGAN paper](https://github.com/LS4GAN/uvcgan)

Important parameters and parameters other than default:

Modify the script to point to your own dataset

Generator: Script- scripts/train/celeba/bert_celeba_preproc-256.py

lr 0.00015625 / Batch_size: 16 Epoch 550

Whole CYCLE Network: Script- scripts/train/selfie2anime/cyclegan_selfie2anime-256.py

lr 0.0001 / Batch Size: 8 Epoch: 150
